# Chinese Adults’ Willingness to Pay for Mandatory Nutrients Reporting on Nutrition Facts Table

**DOI:** 10.3390/nu15234881

**Published:** 2023-11-22

**Authors:** Zeying Huang, Haijun Li, Jiazhang Huang

**Affiliations:** 1 Institute of Food and Nutrition Development, Ministry of Agriculture and Rural Affairs, Beijing 100081, China; huangzeying@caas.cn; 2 School of Information & Intelligence Engineering, University of Sanya, Sanya 572022, China

**Keywords:** Nutrition Facts Table, nutrient, choice experiment, nutrition labeling, preference statement, willingness to pay

## Abstract

The Chinese food industry has opposed the mandatory inclusion of increased nutrients in the Nutrition Facts Table (NFT), thus impeding its improvement. This poses a challenge to the endeavors aiming to assist consumers in cultivating healthy dietary habits that incorporate reduced saturated fatty acids and added sugars while ensuring the adequate intake of essential micronutrients. This study conducted a choice experiment to investigate Chinese consumers’ preference for updated labeling schemes among 630 adults that were randomly selected from Central, North, East, South, Northwest, Southwest, and Northeast China. It revealed that respondents were willing to pay the highest premium for the most mandatory nutrients (22.575% of the food price per unit). Respondents preferred the NFT with the most mandatory nutrients if they met the following population characteristics: female; non-overweight or obese; without a college degree; possessed an annual household disposable income between 50,000 and 99,999 CNY; from North China; lived in rural areas and often cooked for family; cared about food nutrition. Two combinations of NFT information received the highest preference: (1) the NFT detailing the most mandatory nutrients and their content values and nutrient reference values (NRV%); (2) the NFT containing the most nutrients and the nutrients in 100 g/mL or a serving. The first and second combinations attracted a premium of 14.884% and 31.833% of the food price per unit, respectively.

## 1. Introduction

The Nutrition Facts Table (NFT) on prepackaged foods is a nutrition labeling tool for dietary guidance in China. In 2011, the Ministry of Health of China issued the National Food Safety Standard of General Rules for Nutritional Labeling of Prepackaged Foods (GB 28050—2011), which has mandated food manufacturers to label four nutrients—carbohydrate, protein, fat, and sodium—and their content values and nutrient reference values percentage (NRV%) per 100 g/mL of food in the outer package since 2013 [1] (Table 1). However, the nutrient information provided in the NFT is inadequate for guiding the establishment of healthy dietary habits among Chinese residents. Specifically, the current NFT lacks mandatory information on sugar and saturated fatty acid contents and includes information on only one micronutrient. However, in China, in 2019, nearly 20% of children and adolescents aged 6–17 frequently drank sugar-sweetened beverages, their dietary fat supply ratio reached 34.6% (exceeding 1/3 of the daily consumption), and their daily intake of micronutrients was insufficient [2,3].

Currently, the NFTs in the United States, Canada, the United Kingdom, and Australia show 14, 9, 6, and 6 nutrients, respectively [4,5,6,7]. In contrast, only four nutrients are shown in the Chinese NFT, whose public nutrition intervention was weaker than that of the above countries. Increasing the mandatory nutrients in the NFT is an important program proposed by the National Nutrition Plan (2017–2030) and Healthy China Initiative (2019–2030) [8,9]. In 2018, the first draft for public comments of the General Rules for Nutrition Labeling of Prepackaged Food, released by the National Health Commission, proposed the inclusion of saturated fatty acids, sugar, vitamin A, and calcium as mandatory nutrients in the GB 28050 standard [10]; its second round of comments on the draft proposed the inclusion of saturated fatty acids and sugar in 2021 [11]. However, the NFT revision has stalled due to opposition from many food manufacturers, which is rooted in two key reasons. First, all prepackaged food manufacturers would pay more testing fees for the additional mandatory nutrients included in the labeling. Second, consumers’ increased nutrition awareness due to the updated NFT may decrease the market demand for unhealthy or low-nutrient-density foods. Food manufacturers’ support for the updated labeling scheme hinges on whether the revised labels can translate into increased profits. Therefore, our study aims to investigate Chinese consumers’ willingness to pay (WTP) a premium for foods with increased mandatory nutrients in the NFT.

Present studies focus on the comprehension of the NFT [12], factors influencing the labeling use behavior [13,14], and an evaluation of the effectiveness of labeling use [15,16]. With the proposed updated NFT plan in the United States, the influence of the updated NFT on consumers’ understanding of, and attention to, nutrition information, as well as their food purchase intention and behavior, has gradually received attention, reflected by research on increasing the font size of information regarding serving size [17,18], food energy [19], and added sugar content [20,21]. However, not all updated labels were useful. For example, increased serving sizes may lead people to serve larger proportions of food for themselves and others [17], and information regarding added sugar did not change consumers’ choice of beverage [20].

Previous studies pay limited attention to the new mandatory nutrients in the NFT, especially the influence of new mandatory nutrients with other labeling attributes on consumers’ behavior. In addition, relevant research in countries other than the United States is scarce. Therefore, this study aims to explore the influence mechanism of increased mandatory nutrients on consumers’ preference and WTP for foods with NFT in China. The empirical findings offer a solution to the dilemma of updating the NFT and serve as references for countries planning their NFT improvement.

## 2. Hypotheses

Including more mandatory nutrients in the NFT enables consumers to make informed choices favoring high-nutrient-density and healthy foods. This heightened awareness leads consumers to anticipate greater nutritional health benefits from these foods, thus enhancing their preference and WTP for foods with the NFT. Therefore, Hypothesis 1 is proposed below:

**H1.**  *The more nutrients are mandatorily included in the NFT, the stronger the preference and willingness among consumers to pay for those foods.*

The nutrient values in the NFT refer to information such as nutrition content values, nutrients per 100 g/mL or a serving, and the adult’s daily intake of that nutrient. Increased mandatory nutrients show more units of nutrient value, which provides consumers with a more comprehensive understanding of the food’s nutritional value. This may help consumers scientifically control their nutrient intake and enhance their awareness of nutritional and healthy diets, thus improving their preference and WTP for the foods with the NFT. Therefore, Hypothesis 2 is proposed below:

**H2.**  *With more nutrient value units, the increased mandatory nutrients and NFT will enhance consumers’ preference and WTP for such foods.*

The unit of food measurement is used to specify the calculation unit of the total content of nutrients. The more units of food measurement are presented on the NFT, the less time consumers spend converting nutrient content information, so it improves their preference and WTP for more nutritional foods when using the updated NFT. Therefore, Hypothesis 3 is proposed below:

**H3.**  *With more food measurement units, the increased mandatory nutrients will improve consumers’ preference and WTP for such foods when using the NFT.*

## 3. Materials and Methods

### 3.1. Choice Experiment Design

The choice experiment method is particularly effective in examining hypothetical scenarios involving combinations of attributes or characteristics that are not yet available on the market [22]. Since an increase in the nutrients included in the NFT has not been implemented in China, the choice experiment method was adopted in this study. Four attributes and characteristics of the NFT are shown in Table 2. The characteristics of mandatory nutrients are specified by the current GB 28050, the GB 28050 standard text for the first-round comment draft, the GB 28050 standard text for the second-round comment draft, and the NFT in the United States and Canada. The characteristics of the nutrient value units and food measurement units are designed based on the current NFT in China, the United States, England, and Australia. 

A total of 180 (3 × 3 × 4 × 5) combinations of attribute characteristics were obtained via a full-factor design in this study. It was not feasible for respondents to make choices within the 16,110 choice sets (180 × 179/2) if each choice set contained two different NFT schemes. Twelve representative choice sets (see the questionnaire in the Supplementary Materials) were selected from the 16,110 choice sets in the orthogonal design method. An example of the choice sets is shown in Table 3.

### 3.2. Methods

The conditional logit model was used to analyze the effect of different attribute characteristics on consumers’ choice of NFT schemes. This model was developed by McFadden based on the binary logit model [23]. According to the discrete choice theory, consumers choose NFT schemes for their utility maximization, so the random utility brought by an individual choosing a labeling scheme consisting of different attributes is expressed as follows:

(1) $$U_{ij}=ASC+x_{ij}^{\prime }\beta +\varepsilon_{ij}$$

In Equation (1), $x_{ij}^{\prime }$ is a label attribute that varies with individual i (i=1,⋯,I) and scheme j (j=1,⋯,J). β shows the effect of $x_{ij}^{\prime }$ on the random utility $U_{ij}$ but does not depend on the coefficients of scheme j. $\varepsilon_{ij}$ is the random error term. *ASC* is an options-specific constant term used to describe “neither option”. *ASC* = 1 when the consumer selects the “neither option” item; otherwise, *ASC* = 0.

Suppose that individual i believes that the utility brought by scheme j was higher than that of scheme k; the probability of individual i choosing scheme j can be written as follows:

(2) $$\begin{array}{cc} P\left(y_{i}=j|\;x_{ij}\right) & =P\left(U_{ij}\geq U_{ik},\;\forall \;k\neq j\;\right)\\ & =P\left(U_{ik}-U_{ij}\leq 0,\;\forall \;k\neq j\;\right)\\ & =P\left(\varepsilon_{ik}-\varepsilon_{ij}\leq x_{ij}^{\prime }\beta -x_{ik}^{\prime }\beta ,\;\forall \;k\neq j\;\right) \end{array}$$

In Equation (2), $y_{i}=j$ means that individual i chooses NFT scheme j. The random utility brought by $U_{ij}$ and $U_{ik}$ for consumer i to chooses labeling schemes j and k consists of different attributes. $\varepsilon_{ij}$ and $\varepsilon_{ik}$ are random error terms. Assuming that {$\varepsilon_{ij}$} is an independent and identical distribution (IID), Equation (3) can be expressed as follows:

(3) $$P\left(y_{i}=j|\;x_{ij}\right)=\frac{e^{x_{ij}^{\prime }\beta }}{\sum_{k=1}^{J}e^{x_{ik}^{\prime }\beta }}$$

Equation (3) is the conditional logit model. Coefficient β does not depend on the scheme, and there is no need to select the reference scheme and to normalize a part of β to 0 [23]. Individual i’s choice of labeling scheme j is $y_{ij}$, which could be represented by a dummy variable, with 1 representing choice and 0 representing no choice; therefore, the log-likelihood function of the conditional logit model is expressed as:

(4) $$y_{ij}=\ln[\frac{P\left(y_{i}=j|\;x_{ij}\right)}{1-P\left(y_{i}=j|\;x_{ij}\right)}]={\hat{\beta }}_{MLE}x_{ij}^{\prime }$$

In Equation (4), ${\hat{\beta }}_{MLE}$ is the estimated value of the regression coefficient obtained by maximum likelihood estimation. $x_{ij}^{\prime }$ refers to the explanatory variables, such as the mandatory nutrients, nutrient units, units of food measurement, and premium, whether individual i chooses labeling scheme j or not. The model is expressed as follows:

(5) $$y_{ij}=\beta_{i1}X_{ij1}+\beta_{i2}X_{ij2}+\beta_{i3}X_{ij3}+\beta_{i4}X_{ij4}+\varepsilon_{ij}$$

In Equation (5), $X_{ij1},{\;X}_{ij2},{\;X}_{ij3},{\;and\;X}_{ij4}$ indicate the mandatory nutrients, nutrient units, units of food measurement, and premium, respectively. ${\;\beta }_{i1},\;\beta_{i2}$, $\beta_{i3}$, $\beta_{i4}$ represent the marginal utility of the four attributes for individual *i*. $\varepsilon_{ij}$ represents the random disturbance term.

The preference test of consumers regarding commodity attributes could be transformed into a comparison of the WTP for the foods, with each attribute in the NFT corresponding to the utility level of each attribute of the goods for consumers, which could be reflected by the WTP [24].

If ${\;X}_{j1}^{1}=1$ represents more nutrients and $X_{j1}^{0}=0$ represents fewer nutrients, consumers will pay a certain premium for the change in the quantity of nutrients for the unchanged utility. The specific expression is as follows:

(6) $$\begin{array}{c} \beta_{i1}X_{ij1}^{0}+\beta_{i2}X_{ij2}+\beta_{i3}X_{ij3}+\beta_{i4}X_{ij4}+\varepsilon_{ij}\\ =\beta_{i4}(X_{ij4}+{WTP}_{ij1})+\beta_{i1}X_{ij1}^{1}+\beta_{i2}X_{ij2}+\beta_{i3}X_{ij3}+\varepsilon_{ij} \end{array}$$

This can be solved by Equation (6):

(7) $${WTP}_{ij1}=-\frac{\beta_{i1}}{\beta_{i4}}$$

In Equation (7),${\;WTP}_{ij1}$ represents individual i’s WTP for the foods with mandatory nutrients in the labeling. $\beta_{i1}$ and ${\;\beta }_{i4}$ indicate the marginal utility of the attribute of mandatory nutrients and the premium for individual i, respectively. ${WTP}_{ij1}$ > 0 represents that individual i is willing to pay a premium for the labeling with mandatory nutrients; namely, the utility of such an attribute is positive. Similarly, ${\;WTP}_{ij2}$ and ${\;WTP}_{ij3}$ represent individual i’s WTP for the foods with specific nutrient units and units of food measurement in the labeling, respectively.

### 3.3. Collection of Data

The minimum sample size is an important issue that should be considered in the choice experiment. The measurement method proposed by scholars Orme and Johnson et al. [25,26] is adopted, and the calculation formula is as follows:

(8) $$N=500\times \frac{L}{A\times C}$$

In Equation (8), *N* is the minimum sample size, *L* is the maximum attribute characteristics, *A* is the number of options in a choice set, and *C* is the number of choice sets. Calculations showed that this choice experiment required at least 69 samples.

Our proposed questionnaire (see Supplementary Materials) was improved through a pre-survey of 30 adults in Beijing, China. Regarding data availability, a paid online survey service was adopted from Wenjuanxing (https://www.wjx.cn (accessed on 8 September 2022)). Wenjuanxing, a well-known online survey company in China with a member database of 6.2 million registered members of different ages from 31 provinces, mainly provides paid data collection services for its clients. 

People in Northeast China, North China, Northwest China, East China, Central China, Southwest China, and South China have different eating habits [27]. This study randomly selected one province/autonomous region (referred to as ‘province’ hereafter) from the above seven regions, namely Jilin, Inner Mongolia, Shaanxi, Shandong, Henan, Sichuan, and Guangdong, using a stratified random sampling method. In addition to the at least 69 samples requested by Equation (8), this study determined the minimal number of representative random samples (N = 600) in China based on an allowable error of 4% and a confidence level of 95%. We commissioned Wenjuanxing to collect more than 600 adult samples from its member database. From 8th September to 22nd September 2022, Wenjuanxing emailed the questionnaire link to 910 adults that were randomly selected from the above seven provinces, and about 88.13% participated in the online survey. Before data collection, informed written consent was obtained from all participants. Eight CNY, as a cash incentive, was offered to each respondent if their responses were careful and complete. Finally, after data validity was checked, 630 valid samples were used for analysis.

## 4. Results

### 4.1. Descriptive Statistics

As shown in Table 4, the distribution of samples’ gender and residence was similar to that reported in the Main data of the 7th National Population Census in 2020 [28]. This validates the representativeness of the overall sample despite the low proportion of the population aged 60 and above and with a junior high school education and below.

A total of 630 respondents made choice decisions for 24 labeling schemes from 12 choice sets, resulting in a total of 15,120 (630 × 24) valid samples. As shown in Table 5, among the 15,120 valid samples, more than 40% of the respondents chose the given NFT schemes, while only 10.81% did not choose the NFT scheme from each choice set. In addition, 24 choice sets based on the orthogonal experimental design were characterized by uniform dispersion, orderliness, and comparability.

### 4.2. Baseline Regression Results

Stata (17.0, StataCorp LLC, College Station, TX, USA) was used for the correlation test, multicollinearity test, and Brosch–Pagan test of variables before a logit regression analysis was conducted (see Supplementary Material). A significant correlation was found to exist between the independent variable and dependent variables, and the variance inflation factor (VIF) of each independent variable was less than 10, indicating that multicollinearity was not a serious problem. The heteroscedasticity robust standard error was used in regression analysis due to the existence of heteroscedasticity. The attribute characteristics of the NFT were not endogenous to respondents’ choice, since these were exogenously determined in the choice experiment design.

As shown in Table 6, the results of model (1), with the attribute of mandatory nutrients, and model (2), with all attributes of the NFT, showed that the labeling with mandatory nutrients had a positive and significant impact on respondents’ choice, and the most mandatory nutrients had the greatest impact. In addition, the NFT with only NRV%, both per 100 g/mL and per serving, positively affected respondents’ choice, while premium and ASC had a significantly negative influence. Respondents were willing to pay the highest premium for the most mandatory nutrients (i.e., 22.575% of the unit price of foods), followed by the second most nutrients (i.e., 8.250% of the unit price of foods) and food measurement units including both ‘per 100 g/mL’ and ‘per serving’ (i.e., 6.500% of the unit price of foods).

### 4.3. Robustness Test Results

This study tested the robustness of model (2) results by excluding the sample populations who did not often buy prepackaged food, undertake food purchasing or cooking tasks at home, and pay attention to the nutritional value of food in model (3), model (4) and model (5). Table 7 shows that the estimation results of model (2) were robust because the regression coefficients of model (3), model (4), and model (5) were negative, and the odds ratio was similar to the results of model (2).

### 4.4. Heterogeneity Analysis Results

As shown in Table 8, there were differences in the significant influence of including the second most mandatory nutrients and the most mandatory nutrients in the NFT on respondents’ choice of prepackaged foods among all populations except for the elderly group. However, there was almost no significant difference in including the third most mandatory nutrients. Respondents preferred the foods with the second most nutrients and were willing to pay higher premiums if they were female, non-overweight or obese population, an urban resident, from South China, in possession of a college degree and above, an infrequent prepackaged foods’ buyer and often cooked for their family and cared about food nutrition. In comparison, respondents preferred the foods with the most mandatory nutrients in the NFT and were willing to pay the highest premium if they were female, non-overweight or obese population, a rural resident, from North China, without a college degree, an infrequent prepackaged foods’ buyer, and often cooked for their family and cared about food nutrition.

Subgroups combining different gender, age, residence and education levels had a significant WTP for the second most mandatory nutrients and the most mandatory nutrients in the NFT, but these were not significant in the third most mandatory nutrients (see Table 9). The female group under 60 years old, from urban areas, and with a college degree and above were likely to pay a higher premium for the second most mandatory nutrients, while the most mandatory nutrients could attract a higher premium among women with a college degree and above, over 60 years old, and from rural areas.

As can be seen from Table 10, respondents were willing to pay a higher premium for the inclusion of more mandatory nutrients if their annual household disposable income exceeded 10,000 CNY. However, those with an income between 50,000 and 99,999 CNY were willing to pay a higher premium for the inclusion of second most mandatory nutrients and the most mandatory nutrients in the NFT, rather than those with incomes of more than 200,000 CNY.

### 4.5. Analysis Results of Interactive Effects of Attribute Characteristics

Regarding the significant interaction between the nutrient value units and mandatory nutrients, Table 11 shows that respondents tended to choose the foods for which the NFT presented more mandatory nutrients and both nutrient value units (i.e., nutrient content values and NRV) compared to the reference group. The OR ratios of any mandatory nutrients with two nutrient value units were greater than 1. These results suggest that respondents preferred the foods with an NFT labeling both the nutrient content values and NRV.

The probability of respondents choosing the NTF with increased mandatory nutrients, combined with their two units of nutrient value, was 2.011 times higher than that of the reference group. The respondents were willing to pay the highest premium (i.e., 14.884% of the unit price) for the foods labeled with the most mandatory nutrients and two units of nutrient value.

Regarding the interaction of nutrients and food measurement units, Table 12 shows that the odds ratio of the NFT with the most mandatory nutrients, taking ‘per serving’ as the food measurement unit, was the highest, with a probability that was 3.149 times higher than that of the reference group. The respondents were willing to pay the highest premium (i.e., 31.833% of the unit price) for the foods with an NFT showing the most mandatory nutrients, taking ‘per serving’ as the food measurement unit.

## 5. Discussion

### 5.1. Theoretical Contributions

As expected, Hypothesis 1 and Hypothesis 2 were supported. Increased mandatory nutrients in the NFT had a positive and significant effect on consumers’ WTP for the foods, and the nutrient value units could enhance the promotional effect of mandatory nutrients on consumers’ WTP, consistent with the relevant studies [21]. The economic law was illustrated by the present empirical study in China, highlighting that consumers’ WTP for foods with the NFT significantly increased with the inclusion of more mandatory nutrients. The economic law showed that nutrient value units produced an enhanced interaction, enriching the theory of random utility.

Our study revealed that food measurement units could not always improve the positive influence of nutrient quantity on consumers’ choice and WTP. This result did not support Hypothesis 3. One possible reason for this is that the two units of food measurement may be substitutive rather than complementary. More precisely, many choice costs would be paid by consumers, to allow for them choose the right nutritional information for themselves, if they were faced with multiple food units.

The consumers will reduce the choice cost and are more willing to pay for the foods with an intuitive food measurement unit. Therefore, this study took the NFT composed of different attributes as an example to explain that disclosing homogeneous information increases the choice cost. It also makes certain theoretical contributions.

### 5.2. Practical Contributions

The results provided practical guidance for the optimal design of the NFT and food pricing. They revealed that only 10.81% of the respondents did not choose the NFT schemes that were designed, which reflected the consumer demand to update the current NFT.

Moreover, residents’ preference for two types of NFT (i.e., one with the most mandatory nutrients and both the nutrient content values and NRV%, and the other showing the most mandatory nutrients and NRV% and taking ‘per serving’ as the food measurement unit) could assist the government and enterprises in designing the updated NFT. The results also showed that respondents were willing to pay a higher premium for the NFT with more mandatory nutrients, and the extent of their willingness varied across gender, education levels, urban and rural areas, Body Mass Index (BMI) classifications, and household income levels, as well as whether or not they often cook for family, care about food nutrition, and purchase prepackaged foods and their subgroups. These findings provided a basis for food manufacturers to make judgments regarding the establishment of a reasonable premium range and establish differentiated pricing among different population groups. 

### 5.3. Limitations

This study is subject to certain limitations. Firstly, the questionnaire was filled out electronically, potentially leading to less valid responses from elderly individuals and those with a lower level of education due to difficulties with electronic devices and literacy. Secondly, the choice experiment design with a specific food was not conducted to investigate the respondents’ choice regarding the NFT schemes. This could result in some respondents having a limited understanding of the NFT and an inability to accurately assess the value of foods using the NFT. Therefore, future research could adopt an interview questionnaire survey for the elderly population and those with a low level of education. Moreover, the choice experiment with foods commonly consumed by the public as a reference food will be conducted to obtain the respondents’ true willingness to pay.

## 6. Conclusions and Recommendations

This study adopted a representative sample of 630 adults from Central, North, East, South, Northwest, Southwest, and Northeast China to estimate their WTP for the foods with an updated NFT. Our findings revealed that most consumers were willing to choose and pay higher premiums for foods with more mandatory nutrients in NFT. Respondents’ choice and WTP significantly increased with the inclusion of more mandatory nutrients in the NFT, which varied across gender, education levels, household income levels, urban and rural areas, and BMI classifications, as well as whether or not they often cook for family, care about food nutrition, and purchase prepackaged foods and their subgroups. The NFT with the most nutrient content values and NRV%, and the NFT with the most nutrients shown as ‘per serving’ values, were most preferred by consumers and attracted the highest premiums. The following policy recommendations are offered: (1) priority should be given to piloting the NFT with the second most mandatory nutrients and the most mandatory nutrients in population groups such as females with college education and above, the non-overweight or obese population, the middle-income group, those who often cook for family, and those who care about food nutrition. (2) In addition to increasing the mandatory nutrients, the NTF should be updated with the nutrient content values and NRV% as units of nutrient value, and ‘per serving’ as the food measurement unit. 

## Figures and Tables

**Table 1:** The current form of the NFT in China.

Items	Per 100 g/mL or Serving	NRV (Nutrient Reference Values) %
Energy	KJ	%
Protein	g	%
Fat	g	%
Carbohydrate	g	%
Sodium	mg	%

Source: National Food Safety standard of general rules for nutritional labeling of prepackaged foods (GB 28050—2011) [1].

**Table 2:** Attributes and characteristics in the choice.

Attribute	Attribute Characteristics
Mandatory nutrients	(1) Carbohydrates, protein, fat, sodium (2) Carbohydrates, protein, fat, sodium, saturated fatty acids, sugar (3) Carbohydrates, protein, fat, sodium, saturated fatty acids, sugar, vitamin A, calcium (4) Carbohydrates, protein, fat, sodium, saturated fatty acids, sugar, vitamin A, calcium, trans fatty acids, cholesterol, dietary fiber, iron, vitamin C
Nutrient value units	(1) Only the nutrient content values (2) Only NRV% (3) Both the nutrient content values and NRV%
Food measurement units	(1) Per 100 g/mL (2) Per serving (3) Per 100 g/mL and per serving
Premium	(1) 0 Chinese yuan (2) 5% of the unit price of foods (3) 10% of the unit price of foods (4) 15% of the unit price of foods (5) 20% of the unit price of foods

Source: Authors’ own illustration.

**Table 3:** An example of a choice set.

Box 1	Option A	Option B	Option C
Mandatory nutrients	Carbohydrates, protein, fat, sodium	Carbohydrates, protein, fat, sodium, saturated fatty acids, sugar, vitamin A, calcium, trans fatty acids, cholesterol, dietary fiber, iron, vitamin C	
Nutrient units	Both the nutrient content values and NRV%	Only NRV%	
Units of food measurement	Per 100 g/mL and per serving	Per 100 g/mL	
Premium	0 Chinese yuan	10% of the unit price of foods	
I would choose: (Please mark only one box)			

**Table 4:** Sample characteristics.

Characteristics	Items	Samples	Percentage (%)	The 2020 Population Census Data (%)
Gender	Male	315	50	51.24
	Female	315	50	48.76
Age	18~59 years old	616	97.78	86.30
	60 years old and above	14	2.22	13.70
Education level	Primary school or below	4	0.63	8.1
	Junior school	23	3.65	9.5
	Senior school	428	67.94	55.03
	Junior college	154	24.44	24.61
	Postgraduate and above	21	3.33	2.76
Residence	Urban area	378	60	63.89
	Rural area	252	40	36.11

Source: Authors’ own calculation.

**Table 5:** Variables’ description and summary statistics.

Variables	Definition and Assignment	Mean	Standard Deviation	Min.	Max.	Proportion (%)	Obs.
*Dependent*							
Choice	No	—	—	—	—	56.98	8616
	Yes	—	—	—	—	43.02	6504
*Independent*							
Mandatory nutrients	Carbohydrates, protein, fat, sodium	—	—	—	—	25.00	3780
	Carbohydrates, protein, fat, sodium, saturated fatty acids, sugar	—	—	—	—	20.83	3150
	Carbohydrates, protein, fat, sodium, saturated fatty acids, sugar, vitamin A, calcium	—	—	—	—	29.17	4410
	Carbohydrates, protein, fat, sodium, saturated fatty acids, sugar, vitamin A, calcium, trans fatty acids, cholesterol, dietary fiber, iron, vitamin C	—	—	—	—	25.00	3780
Nutrients units	Only the nutrient content values	—	—	—	—	29.17	4410
	Only NRV%	—	—	—	—	37.50	5670
	Both the nutrient content values and NRV%	—	—	—	—	33.33	5040
Units of food measurement	Per 100 g/mL	—	—	—	—	33.33	5040
	Per serving	—	—	—	—	33.33	5040
	Per 100 g/mL and per serving	—	—	—	—	33.33	5040
Premium	%	9.58	6.91	0	20	—	15,120
ASC	No	—	—	—	—	10.81	1634
	Yes	—	—	—	—	89.19	13,486

Source: Authors’ own illustration.

**Table 6:** Baseline regression results.

Independent Variables	Model (1)		Model (2)		
	Coefficient	Odds Ratio	Coefficient	Odds Ratio	WTP
Carbohydrates, protein, fat, sodium (baseline)					
Carbohydrates, protein, fat, sodium, saturated fatty acids, sugar	0.060 (0.052)	1.062 (0.055)	0.022 (0.056)	1.022 (0.057)	0.550 [−2.175, 3.283]
Carbohydrates, protein, fat, sodium, saturated fatty acids, sugar, vitamin A, calcium	0.348 *** (0.046)	1.416 *** (0.065)	0.330 *** (0.049)	1.390 *** (0.069)	8.250 [5.819, 10.663]
Carbohydrates, protein, fat, sodium, saturated fatty acids, sugar, vitamin A, calcium, trans fatty acids, cholesterol, dietary fiber, iron, vitamin C	1.012 *** (0.067)	2.752 *** (0.183)	0.903 *** (0.071)	2.468 *** (0.175)	22.575 [19.100, 26.067]
Only the nutrient content values (baseline)					
Only NRV%	—	—	0.199 *** (0.046)	1.220 *** (0.057)	4.975 [2.700, 7.239]
Both the nutrient content values and NRV%	—	—	0.007 (0.047)	1.007 (0.047)	0.175 [−2.103, 2.470]
Per 100 g/mL (baseline)					
Per serving	—	—	−0.140 *** (0.045)	0.869 *** (0.039)	−3.500 [−5.717, −1.298]
Per 100 g/mL and per serving	—	—	0.260 *** (0.037)	1.297 *** (0.048)	6.500 [4.680, 8.322]
Premium	—	—	−0.040 *** (0.004)	0.960 *** (0.004)	—
ASC	—	—	−2.037 *** (0.103)	0.130 *** (0.013)	−50.925 [−56.002, −45.866]
Log likelihood	−8674.005		−8184.03		
Chi2	375.30 ***		1155.96 ***		
Pseudo R2	0.033		0.087		
Obs.	15,120		15,120		

Note: robust standard errors in parentheses; a 95% confidence interval for WTP in the bracket; *** statistical significant at 1%.

**Table 7:** Robustness test results.

Independent Variables	Model (3)	Model (4)	Model (5)
	Coefficient	Coefficient	Coefficient
Carbohydrates, protein, fat, sodium (baseline)			
Carbohydrates, protein, fat, sodium, saturated fatty acids, sugar	0.038 (0.063)	0.042 (0.065)	0.072 (0.059)
Carbohydrates, protein, fat, sodium, saturated fatty acids, sugar, vitamin A, calcium	0.344 *** (0.055)	0.312 *** (0.057)	0.347 *** (0.053)
Carbohydrates, protein, fat, sodium, saturated fatty acids, sugar, vitamin A, calcium, trans fatty acids, cholesterol, dietary fiber, iron, vitamin C	0.937 *** (0.079)	0.871 *** (0.086)	0.959 *** (0.076)
Control variables	√	√	√
Log likelihood	−6584.827	−5965.961	−7207.768
Chi2	1005.730 ***	784.570 ***	980.40 ***
Pseudo R2	0.088	0.079	0.083
Obs.	12,168	10,824	13,224

Note: robust standard errors in parentheses; *** statistical significant at 1%; √ represents the addition of control variables to the regression equation.

**Table 8:** Heterogeneity analysis results of population groups.

Population Groups	Carbohydrates, Protein, Fat, Sodium, Saturated Fatty Acids, Sugar			Carbohydrates, Protein, Fat, Sodium, Saturated Fatty Acids, Sugar, Vitamin A, Calcium			Carbohydrates, Protein, Fat, Sodium, Saturated Fatty Acids, Sugar, Vitamin A, Calcium, Trans Fatty Acids, Cholesterol, Dietary Fiber, Iron, Vitamin C		
	Coefficient	Odds Ratio	WTP	Coefficient	Odds Ratio	WTP	Coefficient	Odds Ratio	WTP
North China (Obs. = 10,800)	−0.035 (0.067)	0.966 (0.064)	−0.870 [−4.132, 2.393]	0.317 *** (0.059)	1.373 *** (0.081)	7.922 [5.043, 10.800]	0.910 *** (0.085)	2.485 *** (0.212)	22.756 [18.584, 26.929]
South China (Obs. = 4320)	0.167 * (0.101)	1.182 * (0.119)	3.977 [−0.718, 8.672]	0.365 *** (0.092)	1.440 *** (0.132)	8.685 [4.412, 12.958]	0.888 *** (0.129)	2.430 *** (0.314)	21.139 [15.105, 27.174]
Male (Obs. = 7560)	0.094 (0.077)	1.099 (0.084)	1.919 [−1.154, 4.992]	0.323 *** (0.070)	1.381 *** (0.097)	6.583 [3.785, 9.381]	0.952 *** (0.101)	2.590 *** (0.260)	19.421 [15.401, 23.441]
Female (Obs. = 7560)	−0.048 (0.081)	0.953 (0.077)	−1.510 [−6.462, 3.441]	0.340 *** (0.070)	1.405 *** (0.099)	10.628 [6.334, 14.922]	0.860 *** (0.101)	2.363 *** (0.238)	26.879 [20.707, 33.051]
Elderly population (Obs. = 336)	−0.343 (0.477)	0.809 (0.338)	−8.176 [−30.437, 14.084]	0.324 (0.328)	1.383 (0.454)	7.717 [−7.590, 23.023]	0.462 (0.433)	1.588 (0.688)	11.010 [−9.217, 31.237]
Non-elderly population (Obs. = 14,784)	0.029 (0.056)	1.029 (0.058)	0.723 [−2.025, 3.471]	0.329 *** (0.050)	1.390 *** (0.070)	8.237 [5.786, 10.688]	0.912 *** (0.072)	2.490 *** (0.179)	22.808 [19.278, 26.337]
Below bachelor degree (Obs. = 10,920)	−0.011 (0.066)	0.989 (0.065)	−0.276 [−3.487, 2.936]	0.316 *** (0.058)	1.372 *** (0.080)	7.899 [5.037, 10.761]	0.915 *** (0.083)	2.497 *** (0.207)	22.878 [18.825, 26.931]
Bachelor’s degree or above (Obs. = 4200)	0.109 (0.106)	1.115 (0.118)	2.802 [−2.522, 8.126]	0.366 *** (0.093)	1.442 *** (0.134)	9.387 [4.709, 14.066]	0.874 *** (0.140)	2.397 *** (0.335)	22.413 [15.398, 29.428]
Urban area (Obs. = 9072)	0.092 (0.073)	1.097 (0.080)	2.055 [−1.103, 5.213]	0.357 *** (0.064)	1.428 *** (0.091)	8.565 [4.057, 13.073]	0.894 *** (0.093)	2.446 *** (0.228)	19.873 [15.803, 23.942]
Rural area (Obs. = 6048)	−0.081 (0.087)	0.922 (0.080)	−2.397 [−7.417, 2.623]	0.291 *** (0.078)	1.338 *** (0.105)	7.924 [5.137, 10.710]	0.919 *** (0.110)	2.506 *** (0.275)	27.017 [20.681, 33.353]
Overweight and obese (Obs. = 4368)	−0.093 (0.102)	0.912 (0.093)	−1.816 [−5.752, 2.120]	0.244 *** (0.088)	1.276 *** (0.113)	4.779 [1.389, 8.168]	0.848 *** (0.131)	2.336 *** (0.305)	16.636 [11.612, 21.660]
Non-overweight and obesity (Obs. = 10,752)	0.067 (0.066)	1.070 (0.071)	1.870 [−1.741, 5.482]	0.364 *** (0.060)	1.440 *** (0.086)	10.124 [6.882, 13.365]	0.927 *** (0.085)	2.526 *** (0.214)	25.745 [21.126, 30.363]
Often cook for family (Obs. = 12,168)	0.038 (0.063)	1.039 (0.065)	0.959 [−1.724, 5.305]	0.345 *** (0.055)	1.410 *** (0.077)	8.633 [4.801, 14.839]	0.937 *** (0.079)	2.552 *** (0.201)	23.528 [15.894, 35.892]
Do not often cook for family (Obs. = 2952)	−0.043 (0.121)	0.958 (0.116)	−1.015 [−4.504, 8.415]	0.270 ** (0.115)	1.310 ** (0.150)	6.339 [0.720, 21.526]	0.767 *** (0.165)	2.154*** (0.356)	18.016 [7.126, 47.450]
Often care about food nutrition (Obs. = 13,224)	0.072 (0.059)	1.075 (0.064)	1.882 [−0.926, 6.428]	0.347 *** (0.053)	1.415 *** (0.075)	9.076 [5.153, 15.425]	0.959 *** (0.076)	2.608 *** (0.198)	25.050 [17.115, 37.892]
Do not often care about food nutrition (Obs. = 1896)	−0.354 ** (0.163)	0.702 ** (0.115)	−6.232 [−8.258, −1.058]	0.204 (0.144)	0.702 ** (0.115)	3.597 [−0.945, 15.198]	0.505 ** (0.199)	1.657 ** (0.330)	8.889 [1.403, 28.010]
Often buy prepackaged foods (Obs. = 10,824)	0.042 (0.065)	1.043 *** (0.068)	1.071 [−1.750, 5.722]	0.312 *** (0.057)	1.367 *** (0.078)	7.945 [4.085, 14.307]	0.871 *** (0.086)	2.388 *** (0.205)	22.148 [14.353, 34.995]
Do not often buy prepackaged foods (Obs. = 4296)	−0.037 (0.108)	0.963 (0.104)	−0.860 [−4.062, 6.739]	0.374 *** (0.098)	1.454 *** (0.142)	8.599 [2.982, 21.930]	0.991 *** (0.125)	2.694 *** (0.336)	22.775 [12.192, 47.893]

Note: robust standard errors in parentheses; a 95% confidence interval for WTP in the bracket; * *p* < 0.1, ** *p* < 0.05, *** *p* < 0.01.

**Table 9:** Heterogeneity analysis results of the main population subgroups.

Population Subgroups	Carbohydrates, Protein, Fat, Sodium, Saturated Fatty Acids, Sugar			Carbohydrates, Protein, Fat, Sodium, Saturated Fatty Acids, Sugar, Vitamin A, Calcium			Carbohydrates, Protein, Fat, Sodium, Saturated Fatty Acids, Sugar, Vitamin A, Calcium, Trans Fatty Acids, Cholesterol, Dietary Fiber, Iron, Vitamin C		
	Coefficient	Odds Ratio	WTP	Coefficient	Odds Ratio	WTP	Coefficient	Odds Ratio	WTP
Male under 60 years old (Obs. = 7368)	0.106 (0.077)	1.112 (0.086)	2.171 [−0.745, 7.032]	0.324 *** (0.071)	1.383 *** (0.098)	6.644 [3.026, 12.677]	0.974 *** (0.102)	2.647 *** (0.270)	19.961 [12.693, 32.078]
Male over 60 years old (Obs. = 192)	−0.503 (0.694)	0.605 (0.420)	−8.559 [−15.871, 8.577]	0.233 (0.404)	1.263 (0.511)	3.966 [−4.760, 10.257]	0.094 (0.703)	1.098 (0.772)	1.594 [−10.929, 14.713]
Female under 60 years old (Obs. = 7416)	−0.046 (0.082)	0.955 (0.078)	−1.431 [−4.651, 5.635]	0.338 *** (0.071)	1.403 *** (0.099)	10.502 [4.519, 23.626]	0.857 *** (0.102)	2.356 *** (0.240)	26.603 [14.847, 52.391]
Female over 60 years old (Obs. = 144)	−0.293 (0.685)	0.746 (0.511)	−13.084 [−15.209, 9.092]	0.477 (0.688)	1.610 (1.108)	21.260 [15.822, 26.445]	1.090 ** (0.559)	2.975 * (1.664)	48.642 [18.950, 52.036]
Urban male (Obs. = 4800)	0.150 (0.098)	1.162 (0.114)	2.815 [−0.607, 8.925]	0.342 *** (0.086)	1.408 *** (0.122)	6.418 [2.531, 13.360]	0.873 *** (0.128)	2.394 *** (0.306)	16.367 [9.102, 29.341]
Rural male (Obs. = 2760)	−0.004 (0.125)	0.996 (0.124)	−0.091 [−4.067, 11.216]	0.290 ** (0.120)	1.336 ** (0.160)	7.031 [0.901, 24.465]	1.096 *** (0.163)	2.992 *** (0.487)	26.617 [12.755, 66.034]
Urban female (Obs. = 4272)	0.027 (0.108)	1.027 (0.111)	0.759 [−3.610, 12.625]	0.374 *** (0.096)	1.454 *** (0.139)	10.633 [3.632, 29.651]	0.924 *** (0.138)	2.519 *** (0.347)	26.251 [12.717, 63.012]
Rural female (Obs. = 3288)	−0.141 (0.122)	0.869 (0.106)	−4.971 [−8.227, 9.462]	0.299 *** (0.104)	1.349 *** (0.140)	10.582 [2.079, 48.280]	0.783 *** (0.149)	2.189 *** (0.325)	27.686 [10.660, 103.168]
Male below college (Obs. = 5328)	0.083 (0.092)	1.087 (0.100)	1.572 [−1.452, 6.782]	0.326 *** (0.084)	1.385 *** (0.117)	6.159 [2.399, 12.636]	1.048 *** (0.118)	2.853 *** (0.337)	19.812 [12.195, 32.935]
Male with college and above (Obs. = 2232)	0.123 (0.141)	1.131 (0.160)	3.099 [−2.473, 23.642]	0.318 ** (0.126)	1.374 ** (0.173)	8.026 [1.143, 33.403]	0.733 *** (0.190)	2.082 *** (0.395)	18.506 [5.798, 65.361]
Female below college (Obs. = 5592)	−0.097 (0.094)	0.907 (0.085)	−3.227 [−6.418, 5.209]	0.312 *** (0.082)	1.366 *** (0.111)	10.349 [3.479, 28.515]	0.798 *** (0.116)	2.221 *** (0.257)	26.474 [13.056, 61.956]
Female with college and above (Obs. = 1968)	0.096 (0.161)	1.100 (0.177)	2.523 [−3.472, 33.065]	0.425 *** (0.140)	1.529 *** (0.214)	11.194 [2.379, 56.100]	1.046 *** (0.206)	2.847 *** (0.588)	27.572 [10.115, 116.502]

Note: robust standard errors in parentheses; a 95% confidence interval for WTP in the bracket; * *p* < 0.1, ** *p* < 0.05, *** *p* < 0.01.

**Table 10:** Heterogeneity analysis results of population groups with different income levels.

Annual Disposable Family Income Levels	Carbohydrates, Protein, Fat, Sodium, Saturated Fatty Acids, Sugar			Carbohydrates, Protein, Fat, Sodium, Saturated Fatty Acids, Sugar, Vitamin A, Calcium			Carbohydrates, Protein, Fat, Sodium, Saturated Fatty Acids, Sugar, Vitamin A, Calcium, Trans Fatty Acids, Cholesterol, Dietary Fiber, Iron, Vitamin C		
	Coefficient	Odds Ratio	WTP	Coefficient	Odds Ratio	WTP	Coefficient	Odds Ratio	WTP
Less than 10,000 CNY (Obs. = 744)	−0.432 (0.275)	0.649 (0.178)	−20.176 [−27.076, −7.618]	−0.075 (0.237)	0.928 (0.220)	−3.510 [−27.748, 9.493]	0.231 (0.235)	1.260 (0.296)	10.813 [−4.030, 49.319]
10,000~49,999 CNY (Obs. = 2736)	−0.283 ** (0.110)	0.753 ** (0.083)	−8.424 [−9.608, −4.395]	0.117 (0.102)	1.124 (0.115)	3.469 [−1.621, 20.799]	0.776 *** (0.152)	2.172 *** (0.330)	23.071 [9.186, 70.342]
50,000~99,999 CNY (Obs. = 2952)	0.224 * (0.129)	1.251 * (0.162)	5.720 [−0.539, 21.208]	0.418 *** (0.119)	1.519 *** (0.181)	10.687 [3.304, 28.958]	1.291 *** (0.177)	3.638 *** (0.645)	33.009 [16.938, 72.779]
100,000~149,999 CNY (Obs. = 3432)	0.066 (0.114)	1.068 (0.122)	1.356 [−2.331, 9.884]	0.429 *** (0.091)	1.536 *** (0.140)	8.838 [3.688, 20.751]	0.784 *** (0.135)	2.191 *** (0.295)	16.164 [7.684, 35.777]
150,000~199,999 CNY (Obs. = 2784)	0.095 (0.140)	1.100 (0.154)	2.361 [−2.944, 18.744]	0.353 *** (0.124)	1.424 *** (0.177)	8.747 [1.795, 30.217]	0.961 *** (0.184)	2.613 *** (0.480)	23.788 [9.848, 66.837]
200,000 CNY and above (Obs. = 2472)	0.151 (0.147)	1.163 (0.171)	3.245 [−1.943, 19.780]	0.467 *** (0.141)	1.595 *** (0.224)	10.027 [2.700, 33.374]	0.950 *** (0.191)	2.585 *** (0.494)	20.395 [8.112, 59.536]

Note: robust standard errors in parentheses; a 95% confidence interval for WTP in the bracket; * *p* < 0.1, ** *p* < 0.05, *** *p* < 0.01.

**Table 11:** Results of the interaction effect between mandatory nutrients and nutrient value units.

Independent Variables	Coefficient	Odds Ratio	Total Odds Ratio	WTP
Carbohydrates, protein, fat, sodium, saturated fatty acids, sugar	0.207 ** (0.084)	1.230 ** (0.104)		
Carbohydrates, protein, fat, sodium, saturated fatty acids, sugar, vitamin A, calcium	−0.196 *** (0.074)	0.822 *** (0.061)		
Carbohydrates, protein, fat, sodium, saturated fatty acids, sugar, vitamin A, calcium, trans fatty acids, cholesterol, dietary fiber, iron, vitamin C	0.679 *** (0.102)	1.971 *** (0.201)		
Only NRV%	0.345 *** (0.085)	1.413 *** (0.121)		
Both the nutrient content values and NRV%	−0.631 *** (0.085)	0.532 *** (0.045)		
Carbohydrates, protein, fat, sodium, saturated fatty acids, sugar × only NRV%	−0.923 *** (0.111)	0.397 *** (0.044)	0.690	−7.888
Carbohydrates, protein, fat, sodium, saturated fatty acids, sugar × both the nutrient content values and NRV%	0.434 *** (0.131)	1.543 *** (0.202)	1.010	0.221
Carbohydrates, protein, fat, sodium, saturated fatty acids, sugar, vitamin A, calcium × only NRV%	0.271 *** (0.090)	1.312 *** (0.118)	1.524	8.95
Carbohydrates, protein, fat, sodium, saturated fatty acids, sugar, vitamin A, calcium × both the nutrient content values and NRV%	1.351 *** (0.111)	3.861 *** (0.427)	1.688	11.154
Carbohydrates, protein, fat, sodium, saturated fatty acids, sugar, vitamin A, calcium, trans fatty acids, cholesterol, dietary fiber, iron, vitamin C × only NRV%	−0.023 (0.115)	0.977 (0.112)		
Carbohydrates, protein, fat, sodium, saturated fatty acids, sugar, vitamin A, calcium, trans fatty acids, cholesterol, dietary fiber, iron, vitamin C × both the nutrient content values and NRV%	0.651 *** (0.093)	1.918 *** (0.179)	2.011	14.884
Control variables	√	√		
Obs.	15,120	15,120		

Note: robust standard errors in parentheses; ** statistical significant at 5%. *** statistical significant at 1%; √ represents the addition of control variables to the regression equation; the total odds ratio is obtained by multiplying the mandatory nutrients corresponding to the cross-term with the nutrient unit, and the odds ratio of the two.

**Table 12:** Results of the interaction effect between mandatory nutrients and food measurement units.

Independent Variables	Coefficient	Odds Ratio	Total Odds Ratio	WTP
Carbohydrates, protein, fat, sodium, saturated fatty acids, sugar	0.654 *** (0.093)	1.924 *** (0.178)		
Carbohydrates, protein, fat, sodium, saturated fatty acids, sugar, vitamin A, calcium	0.355 *** (0.079)	1.426 *** (0.113)		
Carbohydrates, protein, fat, sodium, saturated fatty acids, sugar, vitamin A, calcium, trans fatty acids, cholesterol, dietary fiber, iron, vitamin C	1.351 *** (0.088)	3.863 *** (0.341)		
Per serving	0.418 *** (0.106)	1.518 *** (0.160)		
Per 100 g/mL and per serving	0.393 *** (0.082)	1.482 *** (0.122)		
Carbohydrates, protein, fat, sodium, saturated fatty acids, sugar × per serving	−1.951 *** (0.138)	0.142 *** (0.020)	0.415	−24.417
Carbohydrates, protein, fat, sodium, saturated fatty acids, sugar × per 100 g/mL and per serving	−0.046 (0.129)	0.959 (0.123)		
Carbohydrates, protein, fat, sodium, saturated fatty acids, sugar, vitamin A, calcium × per serving	−0.085 (0.120)	0.919 (0.110)		
Carbohydrates, protein, fat, sodium, saturated fatty acids, sugar, vitamin A, calcium × per 100 g/mL and per serving	0.042 (0.106)	1.043 (0.110)		
Carbohydrates, protein, fat, sodium, saturated fatty acids, sugar, vitamin A, calcium, trans fatty acids, cholesterol, dietary fiber, iron, vitamin C × per serving	−0.623 *** (0.106)	0.537 *** (0.057)	3.149	31.833
Carbohydrates, protein, fat, sodium, saturated fatty acids, sugar, vitamin A, calcium, trans fatty acids, cholesterol, dietary fiber, iron, vitamin C × per 100 g/mL and per serving	−0.782 *** (0.093)	0.457 *** (0.042)	2.616	26.722
Control variables	√	√		
Obs.	15,120	15,120		

Note: robust standard errors in parentheses; *** statistical significant at 1%; √ represents the addition of control variables to the regression equation; the total odds ratio is obtained by multiplying the mandatory nutrients corresponding to the cross-term with the nutrient unit, and the odds ratio of the two.

## Data Availability

Data are contained within the article.

## Supplementary Materials

The following supporting information can be downloaded at: https://www.mdpi.com/article/10.3390/nu15234881/s1. The supplementary material is the respondent questionnaire.
