# Supplementary material for: Chinese Adults’ Willingness to Pay for Mandatory Nutrients Reporting on Nutrition Facts Table
Source: Nutrients. 2023 Nov 22;15(23):4881. doi: 10.3390/nu15234881 (PMC10708288; doi:10.3390/nu15234881)
Supplement: Supplementary file 1 [file nutrients-15-04881-s001.zip › nutrients-2636404-The questionnaire.pdf]

The questionnaire

**Section 1　Demographic characteristics**

Q1-1 Your gender: A. Male　B. Female

Q1-2 Your birth year: ______

Q1-3 Your height: ______cm

Q1-4 Your weight: ______kg

Q1-5 You are from:______ province/autonomous regions

Q1-6 Your residence: A. Urban area　B. Rural area

Q1-7 Your education level: A. Primary school or below B. Junior school C. Senior school D. Junior college or undergraduate　E. Postgraduate or above

Q1-8 Your household's annual disposable income last year : ______Chinese Yuan

A.Less than 10,000 yuan

B.10,000～49,999 yuan

C.50,000～99,999 yuan

D.100,000～149,999 yuan

E.150,000～199,999 yuan

F.200,000 yuan and above

**Section 2　Food consumption habits**

Q2-1 Are you responsible for food shopping or cooking at home?

A. always

B. often

C. occasionally

D. rarely

E. not at all

Q2-2 Are you concerned about the nutritional value of your food?

A. always

B. often

C. occasionally

D. rarely

E. not at all

Q2-3 Do you buy prepackaged foods?

A. always

B. often

C. occasionally

D. rarely

E. not at all

**Section 3 The choice experiment for eliciting consumers' preference for the nutrition facts table**

Suppose that when you visit the prepackaged foods case in your supermarket during a given month you are presented with two choices of the nutrition facts table on prepackaged foods (Option A and Option B). Label A and Label B have different attributes but we have described below for you. While many attributes vary from Label A to Label B. The following are

descriptions of the attributes but may vary from Label A to Label B:

*Mandatory nutrients.* They are the foods nutrients which are regulated to mark in the nutrition facts table by the government standard.

*Nutrient value units.* They are used to display specific information on mandatory nutrients.

*Food measurement units.* They are used to specify the calculation unit of the total content of nutrients.

*Premium*. It is expressed in percentages of the unit price of foods.

| | Option A | Option B | Option C |
|---|---|---|---|
| Mandatory nutrients | carbohydrates, protein, fat, sodium | carbohydrates, protein, fat, sodium, saturated fatty acids, sugar, vitamin A, calcium, trans fatty acids, cholesterol, dietary fiber, iron, vitamin C | |
| Nutrient value units | both the nutrient content values and NRV% | only the nutrient content values | |
| Food measurement units | per 100 g/mL and per serving | per 100 g/mL | |
| Premium | 0 RMB | 10% of the unit price of foods | |
| I would choose:(Please mark only one box) | | | |

| | Option A | Option B | Option C |
|---|---|---|---|
| Mandatory nutrients | carbohydrates, protein, fat, sodium, saturated fatty acids, sugar | carbohydrates, protein, fat, sodium, saturated fatty acids, sugar, vitamin A, calcium, trans fatty acids, cholesterol, dietary fiber, iron, vitamin C | |
| Nutrient value units | only the nutrient content values | only NRV% | |
| Food measurement units | per 100 g/mL | per serving | |
| Premium | 0 RMB | 20% of the unit price of foods | |
| I would choose:(Please mark only one box) | | | |

| | Option A | Option B | Option C |
|---|---|---|---|
| Mandatory nutrients | carbohydrates, protein, fat, sodium, saturated fatty acids, sugar | carbohydrates, protein, fat, sodium, saturated fatty acids, sugar, vitamin A, calcium, trans fatty acids, cholesterol, dietary fiber, iron, vitamin C | |
| Nutrient value units | both the nutrient content values and NRV% | only the nutrient content values | |
| Food measurement units | per serving | per 100 g/mL and per serving | |
| Premium | 10% of the unit price of foods | 0 RMB | |
| I would choose:(Please mark only one box) | | | |

| | Option A | Option B | Option C |
|---|---|---|---|
| Mandatory nutrients | carbohydrates, protein, fat, sodium | carbohydrates, protein, fat, sodium, saturated fatty acids, sugar | |
| Nutrient value units | both the nutrient content values and NRV% | only NRV% | |
| Food measurement units | per 100 g/mL | per 100 g/mL and per serving | |
| Premium | 10% of the unit price of foods | 5% of the unit price of foods | |
| I would choose:(Please mark only one box) | | | |

| | Option A | Option B | Option C |
|---|---|---|---|
| Mandatory nutrients | carbohydrates, protein, fat, sodium, saturated fatty acids, sugar, vitamin A, calcium | carbohydrates, protein, fat, sodium | |
| Nutrient value units | only the nutrient content values | only NRV% | |
| Food measurement units | per serving | per 100 g/mL | |
| Premium | 0 RMB | 5% of the unit price of foods | |
| I would choose:(Please mark only one box) | | | |

| | Option A | Option B | Option C |
|---|---|---|---|
| Mandatory nutrients | carbohydrates, protein, fat, sodium, saturated fatty acids, sugar | carbohydrates, protein, fat, sodium | |
| Nutrient value units | both the nutrient content values and NRV% | only NRV% | |
| Food measurement units | per 100 g/mL and per serving | per serving | |
| Premium | 20% of the unit price of foods | 15% of the unit price of foods | |
| I would choose:(Please mark only one box) | | | |

| | Option A | Option B | Option C |
|---|---|---|---|
| Mandatory nutrients | carbohydrates, protein, fat, sodium, saturated fatty acids, sugar, vitamin A, calcium | carbohydrates, protein, fat, sodium | |
| Nutrient value units | only NRV% | only the nutrient content values | |
| Food measurement units | per 100 g/mL and per serving | per serving | |
| Premium | 10% of the unit price of foods | 20% of the unit price of foods | |
| I would choose:(Please mark only one box) | | | |

| | Option A | Option B | Option C |
|---|---|---|---|
| Mandatory nutrients | carbohydrates, protein, fat, sodium, saturated fatty acids, sugar, vitamin A, calcium, trans fatty acids, cholesterol, dietary fiber, iron, vitamin C | carbohydrates, protein, fat, sodium, saturated fatty acids, sugar, vitamin A, calcium | |
| Nutrient value units | both the nutrient content values and NRV% | only NRV% | |

| | | | |
|---|---|---|---|
| Food measurement units | per serving | per 100 g/mL | |
| Premium | 5% of the unit price of foods | 20% of the unit price of foods | |
| I would choose:(Please mark only one box) | | | |

| | Option A | Option B | Option C |
|---|---|---|---|
| Mandatory nutrients | carbohydrates, protein, fat, sodium, saturated fatty acids, sugar, vitamin A, calcium, trans fatty acids, cholesterol, dietary fiber, iron, vitamin C | carbohydrates, protein, fat, sodium, saturated fatty acids, sugar,vitamin A, calcium | |
| Nutrient value units | only NRV% | only the nutrient content values | |
| Food measurement units | per 100 g/mL | per 100 g/mL and per serving | |
| Premium | 0 RMB | 15% of the unit price of foods | |
| I would choose:(Please mark only one box) | | | |

| | Option A | Option B | Option C |
|---|---|---|---|
| Mandatory nutrients | carbohydrates, protein, fat, sodium, saturated fatty acids, sugar, vitamin A, calcium | carbohydrates, protein, fat, sodium, saturated fatty acids, sugar, vitamin A, calcium, trans fatty acids, cholesterol, dietary fiber, iron, vitamin C | |
| Nutrient value units | only NRV% | both the nutrient content values and NRV% | |
| Food measurement units | per serving | per 100 g/mL and per serving | |
| Premium | 5% of the unit price of foods | 15% of the unit price of foods | |
| I would choose:(Please mark only one box) | | | |

| | Option A | Option B | Option C |
|---|---|---|---|
| Mandatory nutrients | carbohydrates, protein, fat, sodium | carbohydrates, protein, fat, sodium, saturated fatty acids, sugar, vitamin A, calcium | |
| Nutrient value units | only the nutrient content values | both the nutrient content values and NRV% | |
| Food measurement units | per 100 g/mL and per serving | per 100 g/mL | |
| Premium | 10% of the unit price of foods | 15% of the unit price of foods | |
| I would choose:(Please mark only one box) | | | |

| | Option A | Option B | Option C |
|---|---|---|---|
| Mandatory nutrients | carbohydrates, protein, fat, sodium, saturated fatty acids, sugar | carbohydrates, protein, fat, sodium, saturated fatty acids, sugar, vitamin A, calcium | |
| Nutrient value units | only NRV% | both the nutrient content values and NRV% | |
| Food measurement units | per serving | per 100 g/mL | |
| Premium | 15% of the unit price of foods | 5% of the unit price of foods | |
| I would choose:(Please mark only one box) | | | |
